# Editorial: Wheat disease resistance: diagnosis, germplasm mining, and molecular breeding

**DOI:** 10.3389/fpls.2024.1500414

**Published:** 2024-12-09

**Authors:** Runsheng Ren, Xinli Zhou, Jing Feng

**Affiliations:** ^1^ Institute of Germplasm Resources and Biotechnology/Jiangsu Provincial Key Laboratory of Agrobiology, Zhongshan Biological Breeding Laboratory, Jiangsu Academy of Agricultural Sciences, Nanjing, China; ^2^ Wheat Research Institute, School of Life Sciences and Engineering, Southwest University of Science and Technology, Mianyang, Sichuan, China; ^3^ Institute of Plant Protection, Chinese Academy of Agricultural Sciences, Beijing, China

**Keywords:** diagnosis, germplasm mining, wheat, disease resistance, molecular breeding

As one of the most important food crops in the world, disease resistance in wheat is directly related to global food security and agricultural production efficiency. Breeding wheat for disease resistance combined with good agronomy can potentially improve wheat productivity to meet the future demands. To shed light on the latest breakthroughs and cutting-edge research, Frontiers in Plant Sciences presents this Research Topic: *Wheat Disease Resistance: Diagnosis, Germplasm Mining, and Molecular Breeding*, dedicated to exploring new developments, current challenges, latest discoveries, and future prospects in these fields.

## Diagnosis and genetic basis of wheat disease resistance

Wheat may encounter various diseases during its growth process, such as yellow rust, leaf rust, stem rust, powdery mildew, Fusarium head blight, etc. These diseases not only affect the yield of wheat, but also have a serious impact on its quality (Stukenbrock and Gurr, 2023). Therefore, timely and accurate disease diagnosis is a prerequisite for developing effective prevention and control strategies. The diagnosis of wheat disease resistance mainly includes field natural disease identification, which is the most direct and authentic method to reflect the disease resistance of wheat varieties (Laidig et al., 2021). The evaluation index data can intuitively reflect the resistance ability of wheat varieties to specific diseases. However, this method is greatly influenced by environmental factors such as climate, soil, and cultivation management, and may require years of data accumulation to draw accurate conclusions (Kumar et al., 2019). The diagnosis of wheat disease resistance is still a complex and systematic process. The study conducted by Jevtić and Župunski presented experimental evidence for the case. They conducted a comprehensive analysis of 2715 wheat and wheat related species over a period of 8 years, including phenotypic screening of various diseases including powdery mildew, stripe rust, leaf rust, and stem rust. The findings reveal that the plant reactions to leaf rust and stripe rust infections may be misleading. Because these are heavily influenced not only by prevalent rust races and climatic factors that impact pathogen life cycles but also by variations in the susceptibility reactions of wheat genotypes to the broader agro-ecological conditions.

In order to accurately evaluate the disease resistance of wheat varieties, artificial inoculation is a commonly used method (Francesconi, 2022). Secondly to observe and record the incidence of wheat varieties by controlling the inoculation amount of pathogenic organisms and environmental conditions under laboratory or greenhouse conditions. This method can eliminate the interference of environmental factors and more accurately portray the disease resistance of wheat varieties (Ren et al., 2015; Šarčević et al., 2023). With the development of molecular biology technology, the use of molecular markers and gene detection techniques for diagnosing wheat disease resistance is becoming increasingly important (Jabran et al., 2023; Luo et al., 2023). For example, by detecting the presence of specific disease resistance genes in wheat varieties, and their resistance to a certain disease can be predicted. This method has the advantages of being fast, accurate, and not affected by the environment. In the present Research Topic, Yan et al. identified nine quantitative trait loci (QTLs) and their linked SNP markers for stripe rust resistance. Haile et al. consistently detected six FHB- associated QTL and developed their linked KASP markers in durum wheat. Navathe et al. identified six QTLs conferring resistance to Septoria nodorum blotch and tan spot in wheat. In short, the diagnosis of wheat disease resistance is a complex and systematic process that requires the comprehensive use of multiple technical means and methods. Through accurate disease resistance diagnosis, germplasm resources with excellent disease resistance traits can be screened, providing strong support for disease resistant breeding and ensuring stable and high-yield wheat.

## Exploration of wheat disease resistant germplasm

Exploring the wheat disease resistant germplasm is an important part of wheat breeding work. The materials were widely collected from local wheat varieties, agricultural varieties, wild relatives, and imported germplasm resources from other countries. Methods such as field natural disease identification and artificial inoculation identification were used to identify the disease resistance of germplasm resources and screen out germplasm resources with excellent disease resistance traits. In addition, by utilizing modern biotechnologies such as genomics, transcriptomics, and proteomics, we aim to identify disease resistance genes in wheat and provide genetic resources for disease resistance breeding. In the present Research Topics, Qian et al. screened out nine disease-related genes that show distinctive expression profile after *Bgt* invasion and might serve as potential targets to regulate the resistance against powdery mildew in a resistant durum wheat accession W762. Zhou et al. identified twenty-five candidate genes for *QYrsv.swut-1BL* within the 1.066 Mb region in the resistant cultivar. The discovered disease resistance genes will be utilized through modern biological breeding techniques such as molecular marker assisted selection, genetic modification, gene editing, as well as traditional hybrid breeding, mutagenesis breeding, etc., to create new germplasm with excellent disease resistance traits. Through field experiments and demonstration promotion on the created disease resistant germplasm, their disease resistance effect, yield performance, and quality characteristics in actual production were evaluated. Promote the application of wheat germplasm resources that have been evaluated and confirmed to have excellent disease resistance traits and wide adaptability in production, improve wheat yield and quality, and ensure food security. With the continuous development of biotechnology and innovation in breeding technology, as well as the precision and scale of phenotype identification, the exploration and utilization of wheat disease resistant germplasm will generate more significant results. In the future, it is necessary to continuously strengthen the Research Topic and identification of germplasm resources, and deeply explore and utilize the disease resistance gene resources in wheat. At the same time, we will enhance the research and application of disease resistance breeding technologies to promote the rapid development of wheat disease resistance breeding work.

## Molecular breeding of wheat for disease resistance

Traditional breeding has made significant contributions to the cultivation of wheat varieties and food security worldwide. With the continuous development and innovation of biotechnology, molecular breeding for wheat disease resistance will usher in even broader development prospects. The technical means of molecular breeding for disease resistance mainly include molecular marker assisted selection, transgenic technology, gene editing technology, distant hybridization, and chromosome engineering technology, that can broaden the genetic basis of wheat and increase the level of disease resistance (Wulff and Krattinger, 2022).

## Final considerations

The improvement of wheat disease resistance is a complex and systematic project that requires the comprehensive use of various technical means such as disease diagnosis, germplasm resource exploration and molecular breeding. By strengthening disease monitoring and early warning, deepening the exploration of disease resistant germplasm resources, and actively promoting molecular breeding techniques, it is believed that more significant progress will be made in wheat disease resistance breeding in the future, guaranteeing higher wheat production for needs of the rapidly increasing populations.
